# Factors Affecting Mortality Following Hip Fracture Surgery: Insights from a Long-Term Study at a Level I Trauma Center—Does Timing Matter?

**DOI:** 10.3390/jcm14228104

**Published:** 2025-11-15

**Authors:** Lukas L. Negrin, Thomas Christian, Sandra Kalus, Gyula Kiss, Robin Ristl, Stefan Hajdu

**Affiliations:** 1 Department of Orthopedics and Trauma Surgery, Medical University of Vienna, 1090 Vienna, Austria; thomas.christian@meduniwien.ac.at (T.C.); sandra.kalus@oegk.at (S.K.); gyula.kiss@meduniwien.ac.at (G.K.); stefan.hajdu@meduniwien.ac.at (S.H.); 2 Center for Medical Statistics, Informatics and Intelligent Systems, Medical University of Vienna, 1090 Vienna, Austria; robin.ristl@meduniwien.ac.at

**Keywords:** hip fracture surgery, intertrochanteric fracture, subtrochanteric fracture, femoral neck fracture, Cox regression analysis, independent predictors of mortality, subgroup analyses

## Abstract

**Background/Objectives:** The preoperative length of hospital stay (PLHS) is the only major modifiable factor in hip fracture surgery. Identifying the optimal timing for the procedure is crucial for reducing the risk of death. We aimed to explore the association between PLHS and all-cause mortality within six years among adult trauma care patients, as well as to identify independent predictors of mortality. **Methods:** This retrospective study included all patients ≥ 18 years with primary hip fractures who were admitted to our level I trauma center from 1 January 2015, to 31 December 2018, and who underwent surgery. We subdivided the PLHS into four categories—≤24 h, 24 to 36 h, 36 to 48 h, and >48 h—and performed survival and subgroup analyses. **Results:** The inclusion criteria yielded 1604 females and 700 males, comprising 1235 intertrochanteric and subtrochanteric fractures and 1069 femoral neck fractures. Performing surgery in any of the first three categories was not independently associated with a reduced risk of mortality within six years compared to surgery delayed for more than 48 h. The independent predictors of mortality were older age, male gender, ASA ≥ 3, CCI ≥ 3, in-hospital complications, and a longer postoperative hospital stay. Patients with intertrochanteric fractures had a significantly higher mortality risk compared to those with femoral neck fractures. **Conclusions:** The timing of hip fracture surgery is not an independent predictor of mortality. Surgical delay correlates with mortality, but may reflect comorbidity-related selection rather than a direct causal effect.

## 1. Introduction

Hip fractures represent a significant public health concern worldwide [1]. Their incidence increases sharply with age due to osteoporosis, with over 85% occurring in individuals older than 65 years [2], many of whom experience pre-fracture frailty, functional impairment, and multimorbidity. As the global population is projected to continue growing (end of 2022: 8 billion; 2030: 8.5 billion; 2050: 9.7 billion; 2100: 10.4 billion) [3] along with an increase in life expectancy, the number of hip fractures among the elderly is also expected to rise. This trend will impose a significant socioeconomic burden on public healthcare systems, as almost all patients require surgery.

Hip fractures are associated with all-cause excess mortality, including circulatory and respiratory diseases, mental and behavioral disorders, neoplasms, and nervous system conditions [4]. All-cause excess mortality peaks in the first few months after an injury, gradually decreasing but remaining elevated for over eight years [5]. Patients aged 60 or older with hip fractures are three to four times more likely to die within the first year after the injury than those in the general population [6]. The timing of the surgery is the most crucial factor that can be modified. To reduce costs while ensuring consistently high-quality treatment, governmental authorities have established the preoperative length of hospital stay (PLHS)—defined as the time interval from hospital admission to the start of surgery—as the quality indicator for hip fracture surgery, using thresholds of 24, 36, and 48 h.

In elderly patients, delaying surgery can lead to post-operative delirium, nausea, and loss of appetite. Patients who are confined to bed can lose significant muscle mass daily. However, having surgery within a specified time limit may not be reasonable for every patient. Some individuals may require a surgical delay due to health complications or severe comorbidities. Undergoing surgery too soon could negatively impact their outcomes by limiting the opportunity to address their medical conditions beforehand. Given the conflicting evidence in the literature, the debate regarding a mandatory time limit for hip fracture surgery remains controversial. Our study aimed to examine the association between time to surgery, categorized into four periods, and all-cause mortality over six years for adults representing the patient population in trauma care, while also identifying independent predictors of mortality.

## 2. Materials and Methods

All individuals aged 18 years or older with primary hip fractures who were admitted to our Level I trauma center from 1 January 2015, to 31 December 2018, and who received surgical treatment were included in our retrospective study. The follow-up period for all patients lasted six years or until their death, whichever occurred first. Survival time was calculated from the occurrence of a hip fracture. Mortality indicates death from any cause within six years. The American Society of Anesthesiologists’ physical status classification system (ASA) [7] and the Charlson Comorbidity Index (CCI) [8] were used to assess the patient’s perioperative risk. The time of hospital admission was divided into three periods: (1) weekday core time, from 7:00 am to 3:00 pm, (2) weekday outside core time, and (3) weekends or holidays.

We utilized IBM SPSS Statistics 29 for statistical analysis and visualization. Continuous data are presented as the median, along with the range (in square brackets); nominal data are specified using both numbers and percentages. The Wilcoxon-Mann-Whitney test was utilized to identify significant differences in continuous variables between two groups, while the Kruskal-Wallis test was employed to compare three or more groups. If a significant result was obtained, the Dunn-Bonferroni post hoc test for pairwise comparisons was performed. For categorical data, the chi-square test was applied.

The Cox proportional hazards model was used to calculate hazard ratios (HRs) and adjusted hazard ratios (aHRs), along with 95% confidence intervals (CIs). The assessment of proportionality in the Cox models was conducted using log-minus-log plots of the adjusted survival curves, confirming that the proportionality assumptions were satisfied. We first performed a Cox regression analysis for all patients and subsequently for the subgroups femoral neck, intertrochanteric, subtrochanteric, and combined inter-/subtrochanteric fractures, as well as age < 65/≥65 years and ASA < 3/≥3. To assess the impact of surgery timing on mortality, we examined the following categories: PLHS ≤ 24 h (period I), 24 h < PLHS ≤ 36 h (period II), 36 h < PLHS ≤ 48 h (period III), and PLHS > 48 h (period IV). The reference value used was from period IV. In our model, we included the covariates PLHS category, fracture type, surgical method, time of admission, age, gender, AIS ≥ 3 vs. <3, and CCI ≥ 3 vs. <3, as well as in-hospital complications and length of postoperative hospital stay. After conducting an univariable Cox regression analysis for each covariate, we combined those with significant *p*-values in a multivariable Cox regression analysis.

Additionally, we performed subgroup analyses to compare baseline characteristics and mortality rates in two specific groups: (1) patients with intertrochanteric fractures who were treated with either a dynamic hip screw or a nail, and (2) patients with femoral neck fractures who underwent surgical treatment with screws compared to those who received hemi- or total arthroplasty. Differences in survival rates among subgroups were illustrated using Kaplan–Meier curves, which showed survival over six years following hip fracture. We compared the survival distributions with the log-rank test. In general, analyses were considered significant if *p* < 0.05.

## 3. Results

Our study included 2304 patients (1604 females and 700 males) who underwent surgical treatment for primary hip fractures. Of these, 1235 suffered extracapsular fractures. Specifically, (1) 1043 patients experienced an isolated intertrochanteric fracture, treated with either osteosynthesis (dynamic hip screw, 17; short nail, 969; long nail, 51) or arthroplasty (hemiarthroplasty, 3; total arthroplasty, 3), (2) 85 patients had an isolated subtrochanteric fracture (dynamic hip screw, 1; short nail, 17; long nail, 67), and (3) 107 patients sustained both intertrochanteric and subtrochanteric fractures (short nail, 41; long nail, 66). Among the 1069 patients with femoral neck fractures, osteosynthesis was performed on 309 patients (screws, 130; dynamic hip screw, 172; short nail, 6; long nail, 1), while arthroplasty was conducted on 760 patients (hemiarthroplasty, 638; total arthroplasty, 122).

The median time from admission to surgery was 17 [1, 840] hours. Patients discharged alive from our trauma center (96.4%) had a median stay of 13 [2, 145] days. No participants were lost to follow-up.

**Figure 1:**
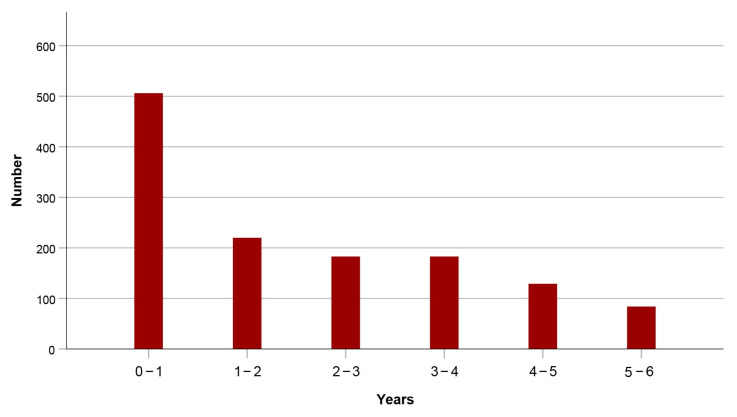
Distribution of fatalities.

During the six-year observation period, 1305 patients (56.6%) died. Figure 1 displays the distribution of fatalities. Notably, 38.8% of those who passed away did so within the first year after sustaining a hip fracture.

### 3.1. Subgroup Analyses Regarding Baseline Characteristics and Mortality Rates

Initially, we divided our study group based on the PLHS. Relevant information is presented and compared in Table 1.

A pairwise comparison revealed that patients who underwent surgery in periods I, II, and III, respectively, experienced significantly shorter postoperative stays (all pairwise *p* < 0.021) and overall hospital stays (all pairwise *p* < 0.001) compared to those treated in period IV. The surgeries performed in periods I and II were significantly shorter than those conducted in period IV (all pairwise *p* < 0.005).

In Table 2, we categorized our patients based on PLHS and age, and performed subgroup analyses comparing survivors and fatalities within each category.

In Table 3, all patients with intertrochanteric fractures, who were treated with osteosynthesis, are categorized according to the implant used. Table 4 displays all patients with femoral neck fractures who underwent screw osteosynthesis or arthroplasty.

### 3.2. Distribution of Patient Ages and Admission Times

**Figure 2:**
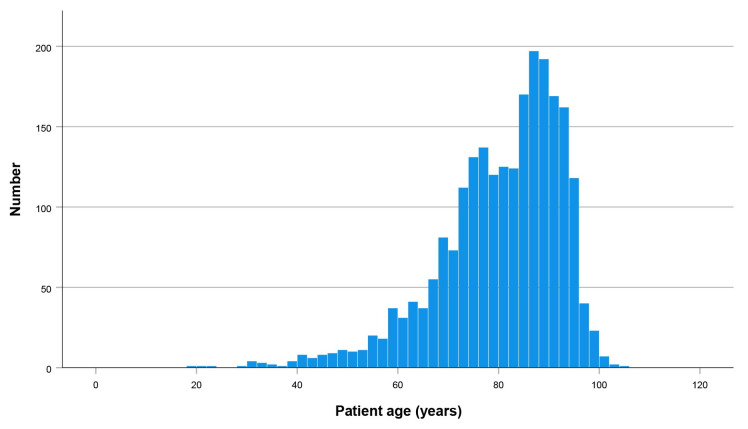
Number of patients by age.

The distribution of patients by age is shown in Figure 2. In the case of hip fractures, elderly patients are typically defined as those aged 65 years or older. To facilitate comparisons, we divided our patients into two groups: those younger than 65 years (252 patients) and those aged 65 years and older (2052 patients).

**Figure 3:**
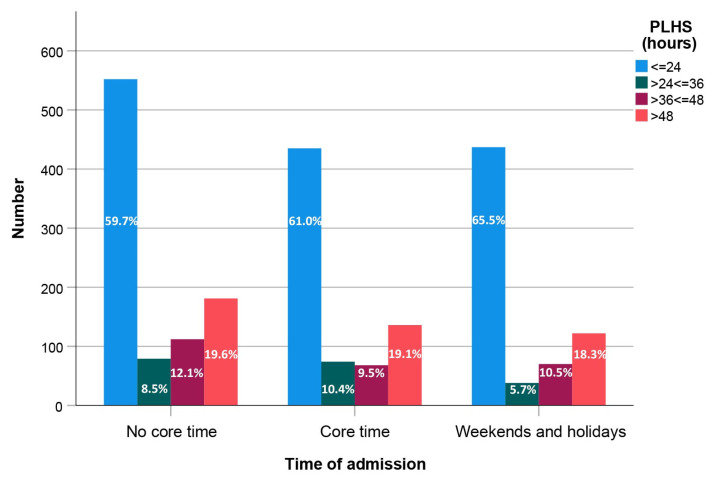
Patient allocation based on admission and surgery timing. The percentages refer to the total number of patients within each group.

Figure 3 shows the timing of patient admissions to our Level I trauma center. During weekday core hours, only 30.9% of patients were admitted.

### 3.3. Kaplan-Meier Curves

**Figure 4:**
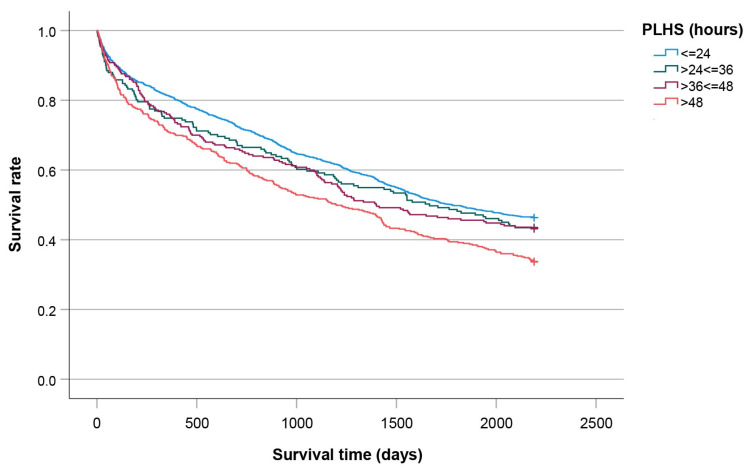
Kaplan-Meier curves plotted for patients categorized by PLHS.

The survival curves defined by the PLHS, as shown in Figure 4, are significantly different (*p* < 0.001). The median survival times were as follows: 1779 days (PLHS ≤ 24), 1666 days (24 < PLHS ≤ 36), 1398 days (36 < PLHS ≤ 48), and 1200 days (PLHS > 48).

**Figure 5:**
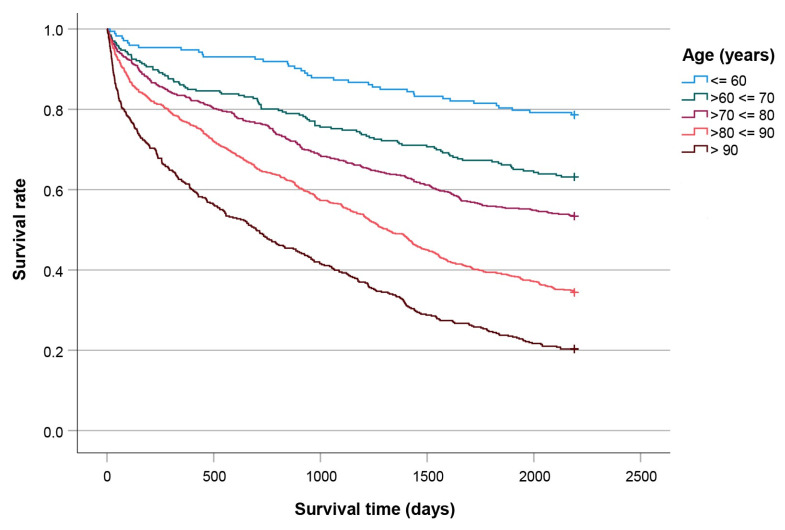
Kaplan-Meier curves plotted for patients categorized by age.

**Figure 6:**
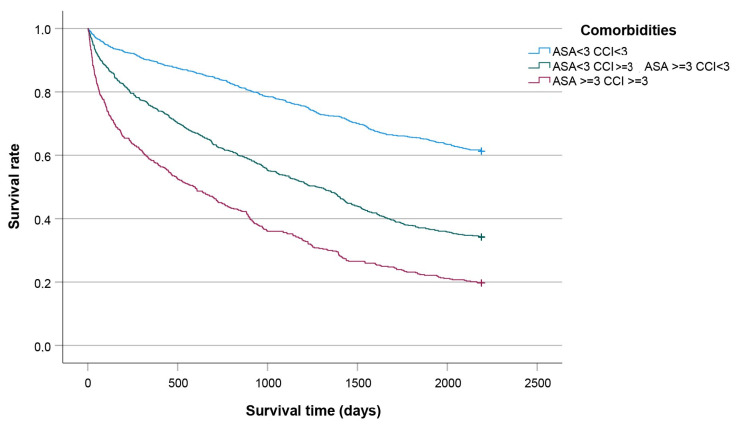
Kaplan-Meier curves plotted for patients categorized by comorbidities.

**Figure 7:**
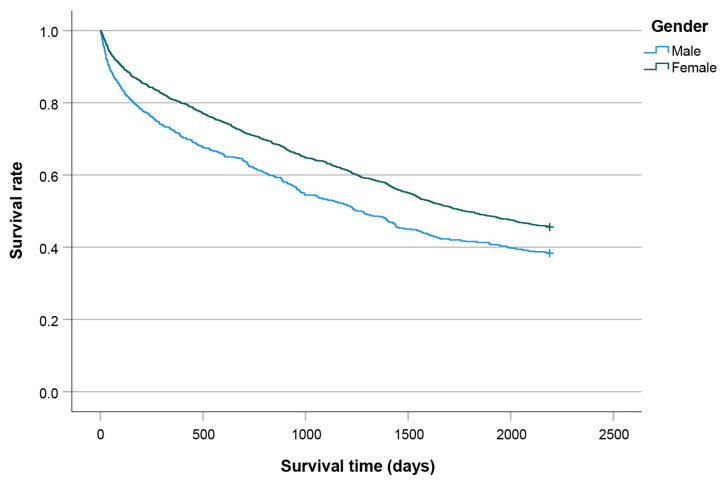
Kaplan-Meier curves plotted for males and females.

**Figure 8:**
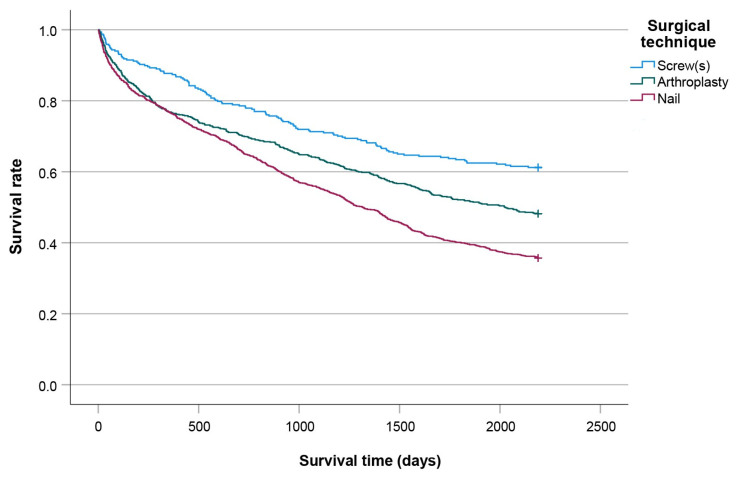
Kaplan-Meier curves plotted according to the surgical technique used.

Figure 5, Figure 6, Figure 7 and Figure 8 show significant differences in survival curves categorized by age, comorbidities, gender, and surgical technique (all *p* < 0.001).

### 3.4. Cox Regression Analyses

Table 5 presents the results of both univariable and multivariable Cox regression analyses for all hip fracture patients, using time from injury to death within six years as the independent variable.

As hip fractures are not a uniform entity, we conducted Cox regression analyses separately for femoral neck, intertrochanteric, subtrochanteric, and concurrent inter- and subtrochanteric fractures (Table 6 and Table 7).

For intertrochanteric fractures, whether isolated or accompanied by a subtrochanteric fracture, we calculated an aHR of 0.626 (95% CI, 0.450–0.871; *p* = 0.05) when comparing PLHS within 24 to 36 h against PLHS exceeding 48 h.

We finally dichotomized our patients into age groups (Table 8) and ASA groups (Table 9) and conducted four separate Cox regression analyses.

## 4. Discussion

In adult patients admitted to our Level I trauma center for hip fracture surgery, the timing of the procedure, whether it occurred within 24 h, between 24 and 36 h, or between 36 and 48 h after the injury, was not found to be independently associated with a reduced risk of mortality compared to procedures that were delayed for more than 48 h. Performing surgery within 24 to 36 h for patients with isolated intertrochanteric fractures resulted in a significantly reduced mortality risk, whereas it led to an increased risk for patients with isolated subtrochanteric fractures. For patients with femoral neck fractures as well as those with combined inter- and subtrochanteric fractures, the time to surgery was not identified as an independent predictor of mortality. The same applies across both age groups (<65 years; ≥65 years) and ASA groups (<3; ≥3). In the entire study population, the factors independently associated with mortality included older age, male gender, an ASA grade of 3 or higher, a CCI grade of 3 or higher, the occurrence of complications during hospitalization, and a longer postoperative hospital stay. Patients with intertrochanteric fractures faced a significantly higher risk of death compared to those with femoral neck fractures.

Meta-analyses are ideal for comparing with our results because they synthesize findings from studies involving patients across different settings, thus deriving results from a diverse population. Unlike us, Klestil et al. [7] and Welford et al. [8] demonstrated the benefits of quickly treating hip fractures. Based on 28 prospective observational studies with data from 31,242 patients, Klestil et al. [7] concluded that undergoing surgery within 48 h of injury was associated with a lower risk of death. Welford et al. [8] analyzed 46 studies, including 521,857 hip fractures. They found that patients treated surgically within 24 h had a reduced mortality risk compared to those operated on after 24 h.

We scanned the literature for studies that analyzed mortality using univariable and multivariable Cox regression analyses and categorized time to surgery (measured in hours rather than calendar days) into distinct periods. We identified a total of 13 studies (Table 10). There is a lack of randomized controlled trials, likely due to ethical concerns about intentionally postponing surgery for some individuals.

We compared our findings to the results of the studies listed in Table 10, all of which centered on hip fractures as a common topic. Unlike our research, one study [16] exclusively examined fragility fractures, while another [13] focused on low-energy trauma. Most of the study populations were composed entirely of patients aged 65 and older [10,11,15,16,17,18,21]. Thus, we did not concentrate exclusively on the entire study population; we also conducted subgroup Cox regression analyses for patients using age 65 as the threshold. However, 18 years [20] (as we did), as well as 50 [12] and 60 years [9,13,19], were also used as a lower age limit, while one study [14] imposed no age restrictions on participants. Since Hongisto et al. [11] studied patients with an ASA grade of 3 or higher, we performed subgroup Cox regression analyses for ASA < 3 and ASA ≥ 3. Five studies reported the median age of their participants. It was either similar to [9,15,20] or identical [14,18] to the median age of 82 years found in our study. Another comparison option was provided by the mortality rates at 30 days and one year following the injury. The 30-day mortality rate varied from 5.1% [9] to 8.2% [20], with our value of 5.2% being close to the lower end. At the one-year mark, the mortality rate ranged from 8% [21] to 30% [17], with our rate of 22% falling in the middle, close to the reported 23.6% [20].

All studies have in common that they compared early surgeries with those performed delayed, although the definition of “delayed” varies. Kristiansson et al. [9] showed an independent association between a PLHS > 48 h and an increased 30-day mortality risk compared to a PLHS ≤ 24 h (aHR = 1.224, *p* < 0.001). When they stratified their patients by ASA classification, the increased mortality risk for a PLHS > 48 h was only observed in patients classified as ASA I and ASA III (aHR = 4.342; *p* < 0.001 and aHR = 1.239; *p* < 0.012). Patients classified as ASA III had a reduced risk of 30-day mortality when surgery was conducted within 36 to 48 h after admission (aHR = 0.880; *p* = 0.044). Öztürk et al. [10] subdivided the PLHS using thresholds of 3, 6, 12, 24, and 48 h. When analyzing the entire study population for 30-day mortality, the authors did not find a significant aHR for any threshold. When stratifying patients by the CCI classification, an independent association with increased 30-day mortality was found for a PLHS > 12 h in those with a CCI grade of 0 (aHR = 1.20; *p* < 0.05) and for a PLHS > 24 h in those with CCI grades of 1 or 2 (aHR = 1.13; *p* < 0.05). Hongisto et al. [11] categorized the PLHS into <12 h, 12 to 24 h, 24 to 48 h, and >48 h. A PLHS of 12 to 24 h (aHR = 8.3; *p* = 0.038) and a PLHS of >48 h (aHR = 11.75; *p* = 0.018) were independently associated with an increased 30-day mortality when compared to a PLHS of <12 h. The only independent predictor of one-year mortality was a PLHS of >48 h (aHR = 2.02; *p* = 0.029). Leer-Salvesen et al. [12] compared the times to surgery of ≤12 h, 13 to 24 h, 25 to 36 h, 37 to 48 h, and >48 h, using the 13 to 24 h delay as the reference category. While no significant aHR was calculated for any time-to-surgery group regarding 30-day mortality, a significant aHR of 1.06 (*p* = 0.003) was observed for the group with surgery performed after 48 h, concerning one-year mortality.

Iida et al. [13] divided their patients into two groups based on the PLHS (≤48 h vs. >48 h). The univariable Cox analysis did not indicate a significant HR for one-year mortality. Kristan et al. [14] conducted a study using a dichotomized PLHS (≤48 h vs. >48 h) as an independent variable, with time from injury to death within one year serving as the dependent variable. A PLHS > 48 h was not found to be an independent risk factor for one-year mortality. Chen et al. [16] focused on three time-to-surgery groups: ≤24 h, 24 to 48 h, and ≥48 h. No significant HR was identified for surgeries performed between 24 and 48 h, or after 48 h, compared to surgeries conducted within 24 h, in univariable regression analysis over a one-year period. Huette et al. [15] also focused on one-year mortality. They reported an aHR of 1.057 (*p* = 0.024) when comparing PLHS > 48 h to PLHS ≤ 48 h. Lund et al. [17] categorized the PLHS into the following time intervals: <12 h, 12 to 23 h, 24 to 47 h, 48 to 71 h, 72 to 95 h, and ≥96 h. The univariable analysis of delayed surgery compared to the reference group PLHS < 12 h showed that postponing surgery was not independently associated with increased one-year mortality. Smektala et al. [18] focused on one-year mortality using a categorized time to surgery (≤12 h, 12 to 36 h, >36 h) as a covariate in multivariable regression analysis. It was not independently associated with one-year mortality. Gdalevich et al. [19] identified an independent association between a time to surgery of ≥48 h and increased one-year mortality (aHR = 1.63; *p* = 0.012). Åhman et al. [20] categorized the time to surgery into ≤12 h, 12 to 24 h, and ≥24 h. The aHR for surgery times of 12 to 24 h and over 24 h in relation to mortality at the most extended follow-up (up to three years) was not statistically significant (reference, ≤12 h). Greve et al. [21] dichotomized the PLHS (≤24 h vs. >24 h). When stratifying their patients by gender, the authors identified an independent association between a PLHS > 24 h and an increased risk of four-month mortality (men, aHR = 1.06, *p* < 0.05; women, aHR = 1.16, *p* < 0.05). When stratifying patients by ASA classification, this association was only significant for those with ASA grades 3 and 4 (aHR = 1.13, aHR = 1.17; *p* < 0.05).

Comparing outcomes is challenging because the authors employed different criteria to define delayed surgery. Depending on the study design, the mortality risk for patients receiving delayed surgical treatment was found to be higher, equal to, or even lower than that of patients who underwent early surgery. Discrepancies may have arisen from various definitions of the time to surgery, which started from the time of injury [12,18,19], admission [9,10,11,13,14,15,17,21], or diagnosis [20], or was not clarified [16]. Survival times varied depending on the starting point of measurements, whether from the injury [13,15,18], surgery [9,10,19], or hospital discharge; in some cases, the starting point was not specified [11,14,17,20,21]. HR was adjusted for various parameters. Covariates such as ASA and CCI grades were categorized differently, which hindered direct comparisons. The outcome of hip fracture surgery may have been affected by the specific hospitals where the procedure was performed. Patients who underwent surgery at high-volume hospitals experienced lower mortality rates compared to those treated at low-volume hospitals [22]. Patients undergoing surgery at rural hospitals faced a 14.6% higher risk of dying compared to those treated at urban non-teaching facilities [23]. Finally, patient selection is crucial. Variations in the characteristics of study populations may explain the differences in mortality risk associated with extended waiting times for surgery.

When analyzing the results of the comparative studies presented in Table 10, without considering the differences in definitions of early and delayed surgery, as well as the varying inclusion criteria, the findings of Öztürk et al. [10] and Leer-Salvesen et al. [12] regarding 30-day mortality are consistent with our results concerning six-year mortality. The same is true for the studies conducted by Iida et al. [13], Kristan et al. [14], Chen et al. [16], Lund et al. [17], Smektala et al. [18], and Åhman et al. [20], which focused on one-year mortality. All seven of these studies suggest that delayed surgery does not increase the risk of death, which matches our findings over a six-year period.

Considering the patient population, the number of comparative studies is reduced to two. Just as we did, Åhman et al. [20] included individuals aged 18 and older, without excluding any specific groups for medical reasons. The authors and we performed Cox regression analyses, using the time to surgery as a covariate subdivided into four groups. Kristan et al. [14] focused on patients without age restrictions, exclusion criteria, or stratification and used a dichotomous PLHS (≤48 h vs. >48 h) as a covariate. Although the studies by Åhman et al. [20] and Kristan et al. [14] and our own research employ different methods, they all show consistent findings: the timing of surgery does not impact mortality in the heterogeneous group of patients that trauma surgeons typically encounter.

We examined patients < 65 years and ≥65 years separately in Cox regression analyses. In both age groups, the categorized PLHS was not found to be an independent predictor of mortality. This aligns with the findings of Öztürk et al. [10], Chen et al. [16], and Lund et al. [17], who focused exclusively on patients aged 65 years and older. Cox regression analyses for patients graded ASA < 3 and those graded ASA ≥ 3 did not show a significant aHR for the PLHS, in contrast to the results reported by Hongisto et al. [11], who studied patients with an ASA grade of 3 or above.

Our study identified age, male gender, ASA ≥ 3, CCI ≥ 3, in-hospital complications, and postoperative length of hospital stay as independent risk factors for mortality; however, confirmation was only found for the first three parameters (age [11,14,15,18,19,20]; male gender [11,18,19,20]; ASA ≥ 3 [19]). Further comparisons with other studies on patients’ health status were not possible due to the differences in the covariates used in the Cox regression analyses. Finally, we found no association between the timing of admission to our Level I center and mortality. The distribution of PLHS categories remained consistent across the three admission time periods, indicating that the PLHS was more influenced by patient factors than by system factors (Figure 3).

The univariable analysis of the four Kaplan-Meier curves related to the PLHS over a six-year period (Figure 4) revealed a statistically significant difference in survival among the groups. This finding aligns with the significant HR identified in the univariable Cox regression analysis. However, confounding by indication may exaggerate the association between early surgery and mortality, as both the timing of surgery and mortality risk are influenced by a patient’s health status. Individuals with more severe medical conditions or comorbidities are more prone to complications and are awaiting optimization, which causes surgical delays. This assumption is supported by the fact that, after adjusting for covariates, the aHR did not achieve statistical significance, indicating no direct relationship between PLHS and mortality. Underlying health issues, rather than the delay, contribute to higher mortality rates.

Our findings suggest that age, ASA ≥ 3, and CCI ≥ 3 are the strongest predictors of mortality in our patient population. In contrast, the aHR related to the categorized PLHS did not reach statistical significance in any of the multivariable Cox regression analyses performed. The ASA system assesses and communicates a patient’s medical comorbidities prior to anesthesia. ASA 3 indicates a patient with severe systemic disease that significantly limits physical activity, ASA 4 refers to a patient with severe systemic disease that poses a constant threat to life, and ASA 5 describes a moribund patient who is not expected to survive without surgical intervention. The CCI assesses comorbidity levels by evaluating both the quantity and severity of 19 predefined comorbid conditions. CCI ≥ 3 indicates a moderate to severe level of comorbidity, meaning the patient has several or serious co-existing health conditions. The primary differences between the ASA score and the CCI are that the ASA score evaluates a patient’s overall health at the time of surgery, whereas the CCI provides insights into a patient’s history of specific diseases and considers age in its scoring. This difference may result in only one of the scores related to comorbidities being predictive of mortality, as shown in Table 7 and Table 8.

The primary limitation of our study is its retrospective design, which necessitated a focus on patient characteristics documented in hospital records. Misclassification of comorbidities may have occurred due to a lack of data on psychiatric conditions other than diagnosed dementia. The documentation did not clarify whether the surgery was postponed due to the patient’s need for medical stabilization or for administrative reasons, such as the availability of medical consultants or the operating theater. As a result, it is impossible to determine the reason for the postponement with certainty in hindsight. Since a pre-fracture functional score was not assessed in our patients, we were unable to determine their frailty level and therefore could not include it as a covariate in the multivariable Cox regression analyses. Finally, data were gathered from a single center, which may limit the generalizability of our findings.

## 5. Conclusions

In summary, surgical delay correlates with mortality but may reflect comorbidity-related selection rather than a direct causal effect. Outcomes are dominated by comorbidities, regardless of timing. Therefore, our results do not endorse a strict guideline. Hip fractures should be treated promptly in healthy patients and in those whose physical status cannot be optimized, as they are at risk of dying during the waiting time. However, surgery should be postponed for patients with serious health issues or significant comorbidities, as they need extensive stabilization before anesthesia and should undergo the procedure as soon as reasonably possible. Surgical treatment of hip fractures should encompass both economic factors and personalized medicine.

## Figures and Tables

**Table 1:** Demographic, clinical, and surgery-related patient characteristics in the study population and the four PLHS subgroups.

		Total	PLHS				*p*-Value
			≤24 h	>24 h, ≤36 h	>36 h, ≤48 h	>48 h	
Patients (*n*)		2304	1424 (61.8%)	191 (8.3%)	250 (10.9%)	439 (19.1%)	
Female		69.6%	71.3%	71.2%	72.4%	62.0%	**0.020**
Age (y)		82 [19, 104]	82 [19, 104]	81 [40, 99]	84 [32, 101]	82 [41, 97]	0.272
ASA ≥ 3		53.2%	43.2%	63.4%	62.8%	75.3%	**<0.001**
CCI ≥ 3		22.5%	16.9%	28.4%	23.9%	37.4%	**<0.001**
Femoral neck fractures		46.4%	39.0%	49.7%	54.4%	64.2%	**<0.001**
Intertrochanteric fractures		45.3%	52.3%	41.9%	38.8%	27.6%	
Subtrochanteric fractures		3.7%	3.7%	4.2%	4.0%	3.2%	
Inter- + subtroch. fractures		4.6%	4.9%	4.2%	2.8%	5.0%	
Additional injury		6.8%	6.8%	6.8%	6.0%	7.1%	0.960
Anticoagulation therapy		48.4%	38.9%	54.3%	64.1%	71.9%	**<0.001**
Anesthesia	Regional	0.3%	0.3%	0.0%	0.4%	0.7%	**<0.001**
	Spinal	59.3%	66.1%	57.4%	51.2%	44.9%	
	General	39.6%	33.6%	42.6%	48.4%	54.1%	
Type of surgery	Screws	5.6%	6.1	4.7	4.4	5.3	**<0.001**
	Dynamic hip screw	8.2%	11.4	4.7	3.2	2.5	
	Short nail	44.7%	50.8	44.0	39.6	28.5	
	Long nail	8.0%	9.3	5.8	5.2	6.4	
	Hemiarthroplasty	27.8%	20.1	33.0	36.8	45.9	
	Total arthroplasty	5.4%	2.3	7.9	10.8	11.4	
Duration of surgery (min)		65 [10, 295]	60 [13, 295]	70 [20, 190]	70 [15, 225]	80 [10, 250]	**<0.001**
Post-surgery hospital stay (d)		12 [0, 145]	12 [1, 145]	12 [0, 92]	12 [1, 78]	13 [0, 78]	**<0.001**
Length of hospital stay (d)		13 [1, 145]	12 [2, 145]	13 [1, 93]	14 [3, 80]	17 [3, 78]	**<0.001**
Stay in the ICU		2.7%	1.3%	4.2%	3.2%	6.9%	**<0.001**
Complications	None	66.4%	69.7%	57.1%	66.4%	59.7%	**0.002**
	Surgical	5.7%	5.4%	6.8%	6.0%	5.9%	
	General	25.3%	22.8%	33.0%	24.4%	30.3%	
	Both	2.8%	2.1%	3.1%	3.2%	4.1%	
In-hospital mortality rate		3.6%	2.7%	7.3%	2.8%	5.5%	**0.001**
30-day mortality rate		5.2%	4.8%	6.8%	4.8%	5.9%	0.592
One-year mortality rate		22.0%	19.0%	25.1%	24.4%	28.9%	**<0.001**
Six-year mortality rate		56.6%	53.7%	56.5%	56.8%	66.3%	**<0.001**

Significant *p*-values are highlighted in bold. h, hours; y, years; min, minutes; d, days.

**Table 2:** Key parameters comparing survivors and fatalities one year after the injury.

	PLHS ≤ 24 h		24 h < PLHS ≤ 36 h		36 h < PLHS ≤ 48 h		PLHS > 48 h	
	Survivor	Fatality	Survivor	Fatality	Survivor	Fatality	Survivor	Fatality
Patients (*n*)	1154	270	143	48	189	61	312	127
Age (y)	81 [19, 104]	87 [45, 101]	79 [40, 98]	89 [66, 98]	83 [32, 98]	87 [55, 101]	80 [51, 97]	87 [41, 97]
	***p* < 0.001**		***p* < 0.001**		***p* = 0.013**		***p* < 0.001**	
ASA ≥ 3	37.7%	67.0%	55.2%	87.5%	58.2%	77.0%	68.6%	92.1%
	***p* < 0.001**		***p* < 0.001**		***p* = 0.008**		***p* < 0.001**	
CCI ≥ 3	12.6%	36.0%	23.1%	44.7%	20.4%	34.4%	30.4%	54.8%
	***p* < 0.001**		***p* = 0.004**		***p* = 0.026**		***p* < 0.001**	
	**60 y < Age** **≤ 70 y**		**70 y < Age** **≤ 80 y**		**80 y < Age** **≤ 90 y**		**Age > 90 y**	
	**Survivor**	**Fatality**	**Survivor**	**Fatality**	**Survivor**	**Fatality**	**Survivor**	**Fatality**
Patients (*n*)	228	38	503	102	633	189	270	168
PLHS	21 [1, 355]	25 [3, 269]	16 [1, 255]	23 [2, 840]	15 [2, 422]	25 [2, 318]	16 [1, 155]	18 [1, 784]
	*p* = 0.646		***p* = 0.042**		***p* < 0.001**		*p* = 0.344	
ASA ≥ 3	39.5%	78.9%	44.7%	85.1%	50.9%	72.3%	58.4%	74.8%
	***p* < 0.001**		***p* < 0.001**		***p* < 0.001**		***p* < 0.001**	
CCI ≥ 3	17.6%	57.9%	19.3%	54.5%	16.5%	36.1%	20.6%	35.4%
	***p* = 0.001**		***p* < 0.001**		***p* < 0.001**		***p* < 0.001**	

Significant *p*-values are highlighted in bold.

**Table 3:** Baseline characteristics of patients with intertrochanteric fractures treated with osteosynthesis.

	Total	Dynamic Hip Screw	Short and Long Nails	*p*-Value
Patients (*n*)	1144	17	1127	
Female	71.6%	58.8%	71.8%	0.240
Age (y)	84 [28, 104]	80 [28, 92]	84 [32, 104]	**0.019**
ASA ≥ 3	54.5%	41.2%	54.7%	0.267
CCI ≥ 3	24.4%	11.8%	24.5%	0.223
Duration of surgery (min)	50 [13, 295]	60 [30, 135]	50 [13, 295]	0.059
PLHS (h)	13 [1, 422]	12 [3, 287]	13 [1, 422]	0.506
Post-surgery hospital stay (d)	12 [0, 145]	12 [5, 21]	12 [0, 145]	0.424
Length of hospital stay (d)	13 [2, 145]	12 [5, 26)	13 [2, 145]	0.586
Stay in the ICU	2.6%	0.0%	2.6%	0.459
In-hospital complications	33.4%	29.4%	33.5%	0.726
In-hospital mortality	4.1%	0.0%	4.1%	0.390
30-day mortality	6.5.%	0.0%	6.5%	0.275
One-year mortality	23.8%	17.6%	23.9%	0.550
Six-year mortality	64.8%	52.9%	65.0%	0.304

Significant *p*-values are highlighted in bold. y, years; min, minutes; h, hours; d, days.

**Table 4:** Baseline characteristics of patients with femoral neck fractures treated by screw osteosynthesis or replacement surgery.

	Total	Screws/DHS	Arthroplasty	*p*-Value
Patients (*n*)	1062	302	760	
Female	67.1%	63.6%	68.6%	0.119
Age (y)	80 [21, 101]	76 [21, 100]	82 [41, 101]	**<0.001**
ASA ≥ 3	51.4%	45.8%	53.6%	**0.023**
CCI ≥ 3	20.3%	16.6%	21.7%	0.062
Duration of surgery (min)	85 [5, 250]	49 [10, 180]	95 [5, 250]	**<0.001**
PLHS (h)	23 [1, 840]	7 [1, 784]	35 [2, 840]	**<0.001**
Post-surgery hospital stay (d)	12 [0, 92]	11 [0, 64]	12 [0, 92]	**<0.001**
Length of hospital stay (d)	13 [1, 93]	12 [3, 64]	14 [1, 93]	**<0.001**
Stay in the ICU	2.9%	1.3%	3.6%	0.052
In-hospital complications	33.5%	26.8%	36.2%	**0.004**
In-hospital mortality	2.8%	1.7%	3.3%	0.147
30-day mortality	3.8.%	2.3%	4.3%	0.118
One-year mortality	20.3%	12.6%	23.4%	**<0.001**
Six-year mortality	48.4%	38.4%	52.4%	**<0.001**

Significant *p*-values are highlighted in bold. y, years; min, minutes; h, hours; d, days.

**Table 5:** Association between covariates and mortality as estimated by Cox regression analyses, unadjusted and adjusted for potential confounders.

Covariates	HR	95% CI		*p*-Value	aHR	95% CI		*p*-Value
		Lower	Upper			Lower	Upper	
PLHS ≤ 24 h vs. PLHS > 48 h	0.703	0.614	0.804	**<0.001**	0.890	0.767	1.033	0.126
24 h < PLHS ≤ 36 h vs. PLHS > 48 h	0.779	0.624	0.971	**0.026**	0.826	0.660	1.035	0.097
36 h < PLHS ≤ 48 h vs. PLHS > 48 h	0.795	0.650	0.971	**0.025**	0.860	0.700	1.057	0.151
Intertrochanteric vs. femoral neck f	1.508	1.344	1.691	**<0.001**	1.406	1.161	1.703	**<0.001**
Subtrochanteric vs. femoral neck f	1.164	0.861	1.574	0.463	1.139	0.804	1.615	0.463
Inter- and subtroch. vs. femoral neck f	1.482	1.151	1.909	**0.002**	1.609	1.193	2.170	**0.002**
Arthroplasty vs. osteosynthesis	0.841	0.747	0.946	**0.004**	1.005	0.819	1.233	0.965
No core time vs. weekend/holiday	0.922	0.808	1.052	0.226				
Core time vs. weekend/holiday	0.970	0.844	1.112	0.664				
No core time vs. core time	1.031	0.897	1.185	0.445				
Age (y)	1.050	1.044	1.057	**<0.001**	1.050	1.044	1.056	**<0.001**
Gender (female vs. male)	0.780	0.695	0.875	**<0.001**	0.596	0.527	0.674	**<0.001**
ASA (≥3 vs. <3)	2.565	2.283	2.881	**<0.001**	1.716	1.501	1.962	**<0.001**
CCI (≥3 vs. <3)	2.427	2.156	2.732	**<0.001**	1.632	1.430	1.861	**<0.001**
In-hospital complications (yes vs. no)	1.417	1.267	1.585	**<0.001**	1.207	1.072	1.357	**0.002**
Postoperative hospital stay (d)	1.015	1.009	1.020	**<0.001**	1.007	1.001	1.013	**0.026**

Significant *p*-values are highlighted in bold; f, fractures; h, hours; y, years; d, days.

**Table 6:** Adjusted Cox regression analysis for singular femoral neck and intertrochanteric fractures.

	Femoral Neck Fractures				Intertrochanteric Fractures			
Covariates	HR	95% CI		*p*-Value	aHR	95% CI		*p*-Value
		Lower	Upper			Lower	Upper	
PLHS ≤ 24 h vs. PLHS > 48 h	0.903	0.727	1.112	0.356	0.872	0.693	1.099	0.246
24 h < PLHS ≤ 36 h vs. PLHS > 48 h	1.040	0.755	1.434	0.809	0.611	0.428	0.872	**0.007**
36 h < PLHS ≤ 48 h vs. PLHS > 48 h	0.913	0.678	1.230	0.549	0.845	0.618	1.156	0.293
Age (y)	1.049	1.039	1.059	**<0.001**	1.048	1.039	1.057	**<0.001**
Gender (female vs. male)	0.637	0.529	0.766	**<0.001**	0.576	0.482	0.689	**<0.001**
ASA (≥3 vs. <3)	1.923	1.549	2.389	**<0.001**	1.612	1.339	1.941	**<0.001**
CCI (≥3 vs. <3)	1.780	1.442	2.196	**<0.001**	1.520	1.265	1.827	**<0.001**
In-hospital complications (yes vs. no)	1.194	0.993	1.436	0.060	1.267	1.073	1.497	**0.005**
Postoperative hospital stay (d)	1.012	1.003	1.020	**0.006**	0.999	0.989	1.009	0.817
Arthroplasty vs. osteosynthesis	1.029	0.827	1.281	0.796				

Significant *p*-values are highlighted in bold; h, hours; y, years; d, days.

**Table 7:** Adjusted Cox regression analysis for subtrochanteric fractures.

	Subtrochanteric Fractures				Both Inter- and Subtrochanteric Fractures			
Covariates	HR	95% CI		*p*-Value	aHR	95% CI		*p*-Value
		Lower	Upper			Lower	Upper	
PLHS ≤ 24 h vs. PLHS > 48 h	1.274	0.517	3.139	0.598	0.721	0.377	1.377	0.321
24 h < PLHS ≤ 36 h vs. PLHS > 48 h	6.421	1.749	25,564	**0.005**	0.648	0.247	1.698	0.377
36 h < PLHS ≤ 48 h vs. PLHS > 48 h	1.843	0.516	6582	0.347	0.481	0.157	1.475	0.200
Age (y)	1.084	1.048	1.122	**<0.001**	1.070	1.037	1.104	**<0.001**
Gender (female vs. male)	0.400	0.171	0.934	**0.034**	0.357	0.189	0.674	**0.002**
ASA (≥3 vs. <3)	3.242	1.322	7.953	**0.010**	1.191	0.672	2.111	0.550
CCI (≥3 vs. <3)	1.376	0.643	2.942	0.411	1.668	0.893	3.116	0.109
In-hospital complications (yes vs. no)	0.941	0.463	1.910	0.865	1.395	0.805	2.417	0.235
Postoperative hospital stay (d)	1.011	0.984	1.038	0.439	1.015	0.997	1.033	0.113

Significant *p*-values are highlighted in bold; h, hours; y, years; d, days.

**Table 8:** Adjusted Cox regression analysis for age subgroups.

	Patients < 65 Years				Patients ≥ 65 Years			
Covariates	aHR	95% CI		*p*-Value	aHR	95% CI		*p*-Value
		Lower	Upper			Lower	Upper	
PLHS ≤ 24 h vs. PLHS > 48 h	0.787	0.383	1.618	0.515	0.963	0.826	1.122	0.627
24 h < PLHS ≤ 36 h vs. PLHS > 48 h	0.251	0.055	1.148	0.075	0.868	0.690	1.090	0.222
36 h < PLHS ≤ 48 h vs. PLHS > 48 h	0.477	0.137	1.660	0.245	0.933	0.757	1.150	0.516
Intertrochanteric vs. femoral neck f	2.473	1.041	5.870	**0.040**	1.514	1.248	1.836	**<0.001**
Subtrochanteric vs. femoral neck f	0.460	0.081	2.607	0.381	1.204	1.210	1.717	0.306
Inter- and subtroch. vs. femoral neck f	1.069	0.122	9.402	0.952	1.638	1.210	2.217	**0.001**
Arthroplasty vs. osteosynthesis	2.215	0.822	5.965	0.116	1.026	0.834	1.262	0.809
Gender (female vs. male)	0.622	0.353	1.097	0.101	0.689	0.609	0.781	**<0.001**
ASA (≥3 vs. <3)	4.409	2.154	9.022	**<0.001**	1.822	1.592	2.085	**<0.001**
CCI (≥3 vs. <3)	1.693	0.822	3.486	0.153	1.536	1.343	1.757	**<0.001**
In-hospital complications (yes vs. no)	0.984	0.535	1.810	0.960	1.270	1.126	1.432	**<0.001**
Postoperative hospital stay (d)	1.028	0.999	1.057	0.060	1.004	0.998	1.010	0.160

Significant *p*-values are highlighted in bold. f, fractures; h, hours; d, days.

**Table 9:** Adjusted Cox regression analysis for ASA subgroups.

	Patients with ASA < 3				Patients with ASA ≥ 3			
Covariates	aHR	95% CI		*p*-Value	aHR	95% CI		*p*-Value
		Lower	Upper			Lower	Upper	
PLHS ≤ 24 h vs. PLHS > 48 h	0.842	0.598	1.187	0.327	0.896	0.757	1.060	0.199
24 h < PLHS ≤ 36 h vs. PLHS > 48 h	0.962	0.592	1.564	0.876	0.805	0.622	1.042	0.099
36 h < PLHS ≤ 48 h vs. PLHS > 48 h	0.896	0.565	1.422	0.642	0.867	0.688	1.093	0.228
Intertrochanteric vs. femoral neck f	2.039	1.433	2.901	**<0.001**	1.171	0.933	1.471	0.596
Subtrochanteric vs. femoral neck f	1.215	0.536	2.623	0.620	1.078	0.726	1.599	0.174
Inter- and subtroch. vs. femoral neck f	2.729	1.675	4.445	**<0.001**	1.187	0.803	1.754	0.710
Arthroplasty vs. osteosynthesis	1.258	0.865	1.828	0.230	0.890	0.697	1.136	0.351
Age (y)	1.072	1.061	1.084	**<0.001**	1.037	1.029	1.045	**<0.001**
Gender (female vs. male)	0.519	0.418	0.644	**<0.001**	0.646	0.556	0.751	**<0.001**
CCI (≥3 vs. <3)	2.088	1.339	3.256	**0.001**	1.574	1.372	1.806	**<0.001**
In-hospital complications (yes vs. no)	1.433	1.161	1.769	**<0.001**	1.133	0.984	1.305	0.083
Postoperative hospital stay (d)	1.014	1.002	1.026	**0.022**	1.004	0.997	1.011	0.292

Significant *p*-values are highlighted in bold. f, fractures; h, hours; y, years; d, days.

**Table 10:** Characteristics of comparative studies.

Study	Design	Facility	Inclusion Criteria	*n*	Demographics	Covariates in Multivariable Analysis
Kristiansson et al. [9] 2020	Retrospective	Tertiary hospital	Age ≥ 60 y	9270	Mean age, 82.6 y 30-day mortality rate, 7.6%	Age, gender, type of surgery
Öztürk et al. [10] 2019	Retrospective	Data base	Age > 65 y	36,552		Age, gender, BMI, type of fracture and surgery, housing condition, marital status, CCI, drug use, anticoagulation
Hongisto et al. [11] 2019	Prospective	Central hospital	Age ≥ 65 y ASA ≥ 3	724	Mean age, 84.1 y one-year mortality rate, 9.1%	Age, gender, previous living arrangements, polypharmacy, type of fracture
Leer-Salvesen et al. [12] 2019	Retrospective	Data base	Age ≥ 50 y	38,754	Mean age, 81.5 y	Age, gender, ASA, type of fracture, and surgery
Iida et al. [13] 2024	Retrospective		Age > 60 y Low-energy trauma	389	Mean age, 84.1 y one-year mortality rate, 10%	
Kristan et al. [14] 2021	Retrospective	Tertiary hospital		641	Median age, 82 y; 2% younger than 40 y 30-day mortality rate, 5.1%; one-year mortality rate, 18.4%	Age, ASA, preinjury residence, surgery type, anticoagulation
Huette et al. [15] 2020	Prospective	Tertiary hospital	Age ≥ 65 y	309	Median age, 85 y one-year mortality rate, 23.9%	Age, ASA, BMI, prefracture status, type of surgery
Chen et al. 2019 [16]	Retrospective	Tertiary hospital	Age > 65 y fragility fractures	313	One-year mortality rate, 10.9%	
Lund et al. [17] 2014	Retrospective	Data base	Age > 65 y	6143	62.2% older than 80 y one-year mortality rate, 30.0%	
Smektala et al. [18] 2008	Prospective	268 acute care hospitals	Age ≥ 65 y	2916	Median age, 82 y one-year mortality rate, 19.7%	Age, gender, ASA, BMI, malignancy, kidney dysfunction, COPD, postoperative complications
Gdalevich et al. [19] 2004	Retrospective	Regional hospital	Age ≥ 60 y	651	49.6% aged 80 y or older one-year mortality rate, 18.9%	Age, gender, ASA, postoperative complications, post-injury mental deterioration
Åhman et al. [20] 2018	Retrospective	Data base	Age ≥ 18 y	14,942	Median age, 83 y 30-day mortality rate, 8.2%; one-year mortality rate, 23.6%	Age, gender, ASA, type, period, and duration of surgery, ICU admission, type of anesthesia
Greve et al. [21] 2020	Retrospective	Data base	Age ≥ 65 y	59,675	Mean age, 83 y 30-day mortality rate, 8%	Age, ASA, type of fracture, and surgery

*n*, patient number; y, years.

## Data Availability

The raw data supporting the conclusions of this article will be made available by the authors upon request.
